# Interactions of Adiponectin and Lipopolysaccharide from *Porphyromonas gingivalis* on Human Oral Epithelial Cells

**DOI:** 10.1371/journal.pone.0030716

**Published:** 2012-02-02

**Authors:** Dominik Kraus, Jochen Winter, Søren Jepsen, Andreas Jäger, Rainer Meyer, James Deschner

**Affiliations:** 1 Department of Prosthodontics, Preclinical Education, and Material Sciences, University of Bonn, Bonn, Germany; 2 Clinical Research Unit 208, University of Bonn, Bonn, Germany; 3 Department of Periodontology, Operative and Preventive Dentistry, University of Bonn, Bonn, Germany; 4 Department of Orthodontics, University of Bonn, Bonn, Germany; 5 Institute of Physiology II, University of Bonn, Bonn, Germany; 6 Experimental Dento-Maxillo-Facial Medicine, University of Bonn, Bonn, Germany; Charité, Campus Benjamin Franklin, Germany

## Abstract

**Background:**

Periodontitis is an inflammatory disease caused by pathogenic microorganisms, such as *Porphyromonas gingivalis*, and characterized by the destruction of the periodontium. Obese individuals have an increased risk for periodontitis and show decreased serum levels of adiponectin. This in-vitro study was established to examine whether adiponectin modulates critical effects of lipopolysaccharide (LPS) from *P. gingivalis* on oral epithelial cells (OECs).

**Methodology/Principal Findings:**

The presence of adiponectin and its receptors in human gingival tissue samples and OECs was analyzed by immunohistochemistry and PCR. Furthermore, OECs were treated with LPS and/or adiponectin for up to 72 h, and the gene expression and protein synthesis of pro- and anti-inflammatory mediators, matrix metalloproteinases (MMPs) and growth factors were analyzed by real-time PCR and ELISA. Additionally, cell proliferation, differentiation and in-vitro wound healing were studied. The nuclear translocation of NFκB was investigated by immunofluorescence. Gingival tissue sections showed a strong synthesis of adiponectin and its receptors in the epithelial layer. In cell cultures, LPS induced a significant up-regulation of interleukin (IL) 1β, IL6, IL8, MMP1 and MMP3. Adiponectin abrogated significantly the stimulatory effects of LPS on these molecules. Similarly, adiponectin inhibited significantly the LPS-induced decrease in cell viability and increase in cell proliferation and differentiation. Adiponectin led to a time-dependent induction of the anti-inflammatory mediators IL10 and heme oxygenase 1, and blocked the LPS-stimulated NFκB nuclear translocation.

**Conclusions/Significance:**

Adiponectin may counteract critical actions of *P. gingivalis* on oral epithelial cells. Low levels of adiponectin, as observed in obese individuals, may increase the risk for periodontal inflammation and destruction.

## Introduction

Periodontitis is a chronic inflammatory disease and characterized by the progressive destruction of the tooth-supporting tissues, i.e. periodontium. The disease is caused by pathogenic bacteria embedded in a biofilm on the tooth surface and leads to epithelial proliferation in combination with periodontal pocket formation, increased tooth mobility and even tooth loss [1]. Based on data from the National Health and Nutrition Examination Survey (NHANES) III, which assessed the health and nutritional status in the United States, it is estimated that half of the US population aged over 30 years suffers from periodontitis [2]. Periodontitis has a significant negative impact on a wide range of physical, psychological and social aspects of quality of life in affected individuals [3], [4]. Furthermore, periodontal infections are associated with cardiovascular diseases, diabetes mellitus, arthritis, preterm low birth weight and other systemic diseases and conditions [5].

Although a variety of microorganisms have been associated with periodontitis, *Porphyromonas gingivalis* represents one of the main etiologic agents in the initiation and progression of periodontal diseases, and is found more frequently and in higher numbers at sites of periodontal inflammation [6]. *P. gingivalis* is characterized by a great number of virulence factors with proteases, such as gingipains, being the most important ones [7]. Another key virulence factor of *P. gingivalis* is lipopolysaccharide (LPS) [8]. LPS is the major macromolecule on the outer surface of gram-negative microorganisms and binds to a Toll-like receptor (TLR) 4–MD-2–CD14 protein complex. However, LPS of *P. gingivalis* is different from those of enterobacteria in that LPS of *P. gingivalis* also signals through TLR2 and is less potent in inducing an inflammatory reaction [8]–[10]. The oral epithelium is the first physical barrier, which periodontopathogenic bacteria encounter [6]. By the release of pro-inflammatory and chemotactic cytokines, matrix-degrading enzymes and prostaglandins, oral epithelial cells, which express TLRs, function as non-professional inflammatory cells and help professional cells of the innate and adaptive immune system to clear the bacterial infection [11]–[13]. Therefore, oral epithelial cells can actively participate in periodontal inflammation. However, a balance between pro- and anti-inflammatory mediators is critical. If the inflammatory response is exaggerated, irreversible loss of periodontal tissues occurs [14], [15]. Levels of pro-inflammatory cytokines, such as interleukin (IL) 1β, IL6, and IL8, which promote recruitment and activation of professional inflammatory cells, are increased at inflamed sites [16]–[18]. Although anti-inflammatory mediators, such as IL10, antagonize the pro-inflammatory activities, the balance between pro- and anti-inflammatory mediators is shifted towards inflammation [14], [15]. In response to pathogenic microorganisms and inflammatory mediators, resident cells and infiltrated inflammatory cells of the periodontium release enzymes, such as matrix metalloproteinases (MMPs). MMPs can cleave all components of the extracellular matrix and, additionally, activate, deactivate, or modify non-matrix bioactive molecules [19]. In periodontitis, the balance between matrix synthesis and degradation is disrupted and shifted towards periodontal tissue destruction [19].

As mentioned above, periodontitis has been shown to affect systemic health. Conversily, systemic diseases, such as diabetes mellitus, can also increase the risk of periodontitis. Recent meta-analyses have revealed that obesity is also significantly associated with periodontitis, and it has been suggested that adipokines, such as adiponectin, which are cytokines released from the adipose tissue, might be a critical pathomechanistic link in this association [20]–[22]. Adipokines not only regulate food intake and energy expenditure, but also immune, inflammatory and wound healing processes. For example, adiponectin plays an anti-inflammatory role in the pathophysiology of several chronic inflammatory diseases [23], [24]. Heme oxygenase 1 (HMOX1) has recently been identified as an important downstream mediator for the anti-inflammatory actions of adiponectin in primary cultures of Kupffer cells [25]. HMOX1 catabolizes heme into carbon monoxide, biliverdin and free iron, which then exert the anti-inflammatory, anti-apoptotic and anti-proliferative effects of HMOX1 [26]. Besides its effects on inflammatory mediators, adiponectin has also been shown to be involved in cell proliferation and differentiation [27]. A common marker of keratinocyte differentiation is involucrin, which first appears in the cytoplasm of differentiating cells in the stratum spinosum and is then cross-linked to membrane proteins in the stratum granulosum by the action of transglutaminase enzymes, which provides structural support to the cells [28]–[30].

Since serum levels of adiponectin have been shown to be negatively correlated with overweight and obesity, the beneficial effects of adiponectin are significantly reduced in overweight and obese individuals [31]. Whether adiponectin regulates the function of periodontal cells and inflammatory processes in periodontitis is as yet unknown. This in-vitro study was established to examine whether adiponectin inhibits the response of oral epithelial cells to LPS from *P. gingivalis* and, thereby, to test the hypothesis that decreased levels of adiponectin may confer an increased risk of periodontal destruction.

## Materials and Methods

### Isolation and culture of human oral epithelial cells

Healthy gingival tissues were obtained from six donors, who underwent tooth extraction and/or dentoalveolar surgery. Written informed parental consent and approval of the Ethics Committee of the University of Bonn were obtained. The gingival specimens were washed twice with phosphate buffered saline (PBS; PAA Laboratories, Cölbe, Germany) supplemented with 1% antibiotic and antimycotic solution (PAA Laboratories) and subsequently digested with collagenase 2 solution (PAA Laboratories) at 37°C for 2.5 h. Afterwards, the epithelium was mechanically separated from the underlying connective tissue layer with sterile forceps. In order to obtain a single cell suspension, the epithelium was then dissociated with 0.05% trypsin in 0.02% EDTA solution (PAA Laboratories) at 37°C for 15 min. After centrifugation, epithelial cells were resuspended in serum-free keratinocyte growth medium (KGM2; Promocell, Heidelberg, Germany) supplemented with 1% antibiotic and antimycotic solution, and grown at 37°C in a humidified atmosphere of 5% CO_2_. Cells were used at passage three and medium was changed every other day. For evaluation of cell viability, epithelial cells were stained with trypan blue solution (Sigma-Aldrich, Munich, Germany) and, subsequently, live and dead cells were counted.

### Cell stimulation

Cells were stimulated with ultrapure LPS from *P. gingivalis* (2 µg/ml; Cayla-InvivoGen, Toulouse, France) and/or recombinant human full-length adiponectin (1 µg/ml; R&D Systems, Wiesbaden, Germany) for up to 72 h. The concentrations applied were based on the results from other studies to ensure that data are comparable [11], [13], [32]. Furthermore, pre-experiments showed that these concentrations had a significant stimulatory effect on pro- and anti-inflammatory cytokines and, therefore, allowed to examine the interactions of LPS and adiponectin on epithelial cells (data not shown).

### Reverse transcription-PCR

Total RNA was isolated using the RNeasy Protect Mini Kit (Qiagen, Hilden, Germany) and quantified using the NanoDrop®ND-1000 Spectrophotometer (NanoDrop Technologies, Wilmington, DE, USA). A total of 1 µg of RNA was reversely transcribed using iScript™ Select cDNA Synthesis Kit (Bio-Rad Laboratories, Munich, Germany) with oligo(dT)-primers according to the manufacturer's protocol. The gene expression of adiponectin and its receptors AdipoR1 and AdipoR2, IL1β, IL6, IL8, IL10, heme oxygenase 1 (HMOX1), MMP1, MMP3, transforming growth factor (TGF) β1, keratinocyte growth factor (KGF), and involucrin was detected by real-time PCR using the iCycler iQ detection system (Bio-Rad Laboratories), SYBR Green (Bio-Rad Laboratories), and specific primers (Metabion, Martinsried, Germany). Primer sequences, annealing temperatures and efficiencies are presented in Table 1. Real-time PCR was performed by adding 50 ng cDNA to a master mix containing primers and iQ™ SYBR® Green Supermix (Bio-Rad Laboratories). Cloned PCR-products derived from the specific primers served as positive controls, whereas water was used as negative control. PCR conditions were defined as follows: 5 min preceding denaturation step at 95°C was succeeded by 50 cycles of 15 s at 95°C, 30 s at annealing temperatures specific for the primers, and 30 s at 72°C for elongation. Relative differential gene expression was calculated using the method described by Pfaffl [33] with β-actin serving as house-keeping gene. Additionally, the PCR products for adiponectin, AdipoR1 and AdipoR2 were visualized by 2% agarose gel electrophoresis and ethidium bromide staining.

**Table 1:** Sequences, efficiency and annealing temperature of primers.

Gene		Primer sequences	Efficiency	Annealing temperature
Adiponectin	senseantisense	5′-GCCTCTTCAAGAAGGACAAGGCTATG -3′ 5′-CAGTTGGTGTCATGGTAGAGAAG -3′	1.93	69°C
AdipoR1	senseantisense	5′-ACTGGAGCTGGCCTTTATGCTGC -3′ 5′-AGAGAAGGGTGTCATCAGTACAGC -3′	2.04	69°C
AdipoR2	senseantisense	5′-CCATAGGGCAGATAGGCTGGTTGA -3′ 5′-CAGTGCATCCTCTTCACTGCAGC -3′	1.97	69°C
β-actin	senseantisense	5′-CATGGATGATGATATCGCCGCG-3′ 5′-ACATGATCTGGGTCATCTTCTCG-3′	1.84	69°C
HMOX1	senseantisense	5′-CCAGGCAGAGAATGCTGAGTTCAT-3′ 5′-CCGTACCAGAAGGCCAGGTCC-3′	1.97	69°C
IL1β	senseantisense	5′-ATGGCAGAAGTACCTGAGCTCGC-3′ 5′-TTAGGAAGACACAAATTGCATGGTG-3′	1.83	68°C
IL6	senseantisense	5′-ATGAACTCCTTCTCCACAAGC-3′ 5′-CTACATTTGCCGAAGAGCCC-3′	2.12	68°C
IL8	senseantisense	5′-ATGACTTCCAAGCTGGCCGTGG-3′ 5′-TGAATTCTCAGCCCTCTTCAAAAAC-3′	2.02	68°C
IL10	senseantisense	5′-TTAAGGGTTACCTGGGTTGC-3′ 5′-GCCTTGCTCTTGTTTTCACA-3′	1.94	65°C
MMP1	senseantisense	5′-ATGCACAGCTTTCCTCCACTGC-3′ 5′-CACTGGGCCACTATTTCTCCGC-3′	2.05	69°C
MMP3	senseantisense	5′-ATCGATGCAGCCATTTCTGATAAGG-3′ 5′-TCAACAATTAAGCCAGCTGTTACT-3′	2.06	69°C
Involucrin	senseantisense	5′-CCCAGCAACACACACTGCCAGT-3′ 5′-GCTCAGGCAGTCCCTTTACAGCA-3′	1.98	69°C
TGFβ1	senseantisense	5′-GAGCCCTGGACACCAACTAT-3′ 5′-GACCTTGCTGTACTGCGTGT-3′	1.94	69°C
KGF	senseantisense	5′-AGTTGGAATTGTGGCAATCA-3′ 5′-CCGTTGTGTGTCCATTTAGC-3′	1.84	65°C

### ELISA

The concentrations of IL1β, IL8 and MMP1 in culture supernatants at 24 h and 72 h were analyzed by a commercially available enzyme-linked immunoassay (ELISA) kit (RayBiotech, Norcross, USA) according to the manufacturer's instructions. The absorbance was measured with a microplate reader (Bio-Rad Laboratories) at 450 nm. The data were normalized by the cell number, which was measured with an automatic cell counter (Moelab, Hilden, Germany).

### Proliferation assay

The epithelial cell proliferation was determined by using the PromoKine XTT Cell Proliferation Kit (Promocell). Following stimulation with LPS and/or adiponectin for 24 h and 48 h, cells were incubated with XTT reaction solution for 4 h. Finally, the absorbance was measured by using a microplate reader at 490 nm.

### In-vitro wound healing assay

In order to study the wound fill rate in vitro, we used an established in-vitro wound healing model [34], [35]. Briefly, epithelial cells were seeded onto 35 mm culture dishes (Greiner Bio-One, Frickenhausen, Germany) and grown until confluence. Then, defined cell-free areas were created by disrupting the monolayers with sterile instruments in a standardized manner. Subsequently, medium was changed and cells were stimulated, as described above. Pictures of the wounded area were taken on an inverted microscope (Axiovert 25 C, AxioCam, Zeiss, Oberkochen, Germany) immediately after wounding and at 1 d, 2 d and 3 d. The wound areas were then measured and analyzed with the freely available image-processing software ImageJ 1.43 (http://rsb.info.nih.gov/ij). In order to determine the percentage of wound fill, the recovered wound areas at 1 d, 2 d, and 3 d were related to the wound areas measured at baseline.

### Immunohistochemistry

Gingival tissues were derived from patients who underwent wisdom tooth extraction in the Dept. of Oromaxillofacial Surgery of the University of Bonn. The tissue samples were fixed in 4% buffered formalin (Sigma-Aldrich) and embedded in paraffin. In order to assess the presence of adiponectin, 4 µm thick tissue slices were prepared and immunostaining for adiponectin in the epithelial and subepithelial layers of the gingiva was performed. Briefly, selected sections were deparaffinized, rehydrated and rinsed with tris-buffered saline (TBS, Sigma-Aldrich) for 10 min. Endogenous peroxidase was blocked in a methanol/H_2_O_2_ (Merck, Darmstadt, Germany) solution in the dark for 45 min. Sections were blocked with 1% bovine serum albumin (BSA, Sigma-Aldrich) at room temperature (RT) for 20 min and incubated with rabbit anti-adiponectin (Santa Cruz Biotechnology, Heidelberg, Germany) in a 1∶50 dilution in a humid chamber overnight. Antigen–antibody binding was visualized using the EnVision Detection System Peroxidase/DAB (Dako, Hamburg, Germany) and slides were counterstained with Mayer's haematoxylin.

### Immunofluorescence

Oral epithelial cells were grown on coverslips and cultured for 24 h. Cells were fixed in 4% paraformaldehyde (Sigma-Aldrich) for 15 min, washed in PBS and treated with PBS containing 0.1% Triton X-100 (Sigma-Aldrich) for 15 min. Then, cells were washed, blocked with 10% BSA for 1 h at RT and incubated with a rabbit anti-adiponectin antibody (Santa Cruz Biotechnology) in a 1∶75 dilution. After washing, cells were incubated with a Cy3-conjugated goat anti-rabbit IgG secondary antibody (1∶500; Dianova, Hamburg, Germany) at RT for 1 h. For nuclear staining, cells were treated with DAPI for 5 min. Finally, slides were washed and mounted with Mowiol/DABCO (Roth, Karlsruhe, Germany) for fluorescence microscopic analysis. Cells treated with LPS and/or adiponectin for 2 h were processed in the same manner, except incubation with a rabbit anti-NFκB (p65) antibody (1∶100; BioLegend GmbH, Fell, Germany). For quantification, all nuclei were counted in five fields of vision at a primary magnification of 40×. NFκB positive nuclei were plotted as percentage of all nuclei.

### Statistical Analysis

Mean ± SEM (n = 6) were calculated. One-way ANOVA and the post-hoc Tukey's multiple comparison test were applied using a statistical software program (GraphPad Software, San Diego, CA, USA). P-values less than 0.05 were considered to be statistically significant.

## Results

### Presence of adiponectin in oral epithelial cells

**Figure 1:**
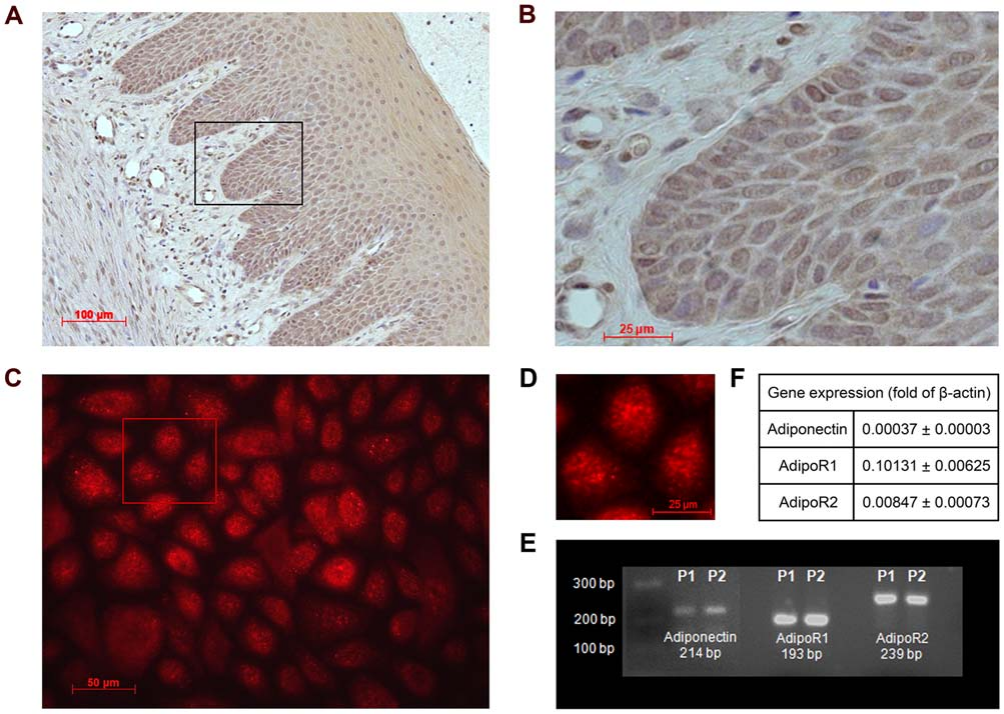
Adiponectin in gingival tissues and cultured oral epithelial cells. (A) Representative gingival tissue section from one out of 6 patients exhibiting adiponectin distribution assessed by immunohistochemistry with DAB-staining (brown color). (B) Higher magnification of the rectangular area shown in (A). (C) Immunofluorescence staining of adiponectin in cultured oral epithelial cells from one out of 6 patients. (D) Higher magnification of the rectangular area shown in (C). (E) Gene expression of adiponectin and its receptors AdipoR1 and AdipoR2 from oral epithelial cells of 2 patients (P1 and P2), as demonstrated by gel electrophoresis. (F) Gene expression of adiponectin and its receptors AdipoR1 and AdipoR2 from oral epithelial cells from 6 patients, as demonstrated by real-time PCR.

Healthy human gingival tissue sections showed a strong adiponectin synthesis in oral epithelial cells, as analyzed by immunohistochemistry. By contrast, only weak staining was found in the subepithelial layer (Fig. 1A and B). The presence of adiponectin in oral epithelium was also confirmed at transcriptional and protein levels in cultured oral epithelial cells, as shown in Fig. 1C–F. Furthermore, oral epithelial cells expressed both receptors for adiponectin (Fig. 1E and F). Taken together, these findings suggest that epithelial cells are a significant source and target of adiponectin in gingival tissues.

### Actions of adiponectin on pro- and anti-inflammatory mediators in oral epithelial cells

**Figure 2:**
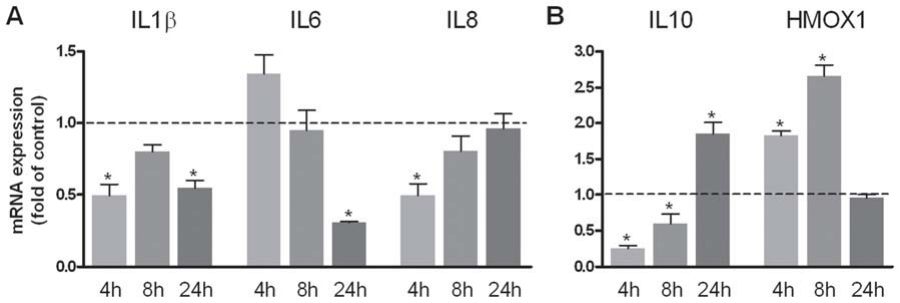
Effects of adiponectin on pro- and anti-inflammatory mediators. Effects of adiponectin (1 µg/ml) on IL1β, IL6 and IL8 (A) as well as IL10 and HMOX1 (B) in oral epithelial cells at 4 h, 8 h, and 24 h. Mean ± SEM; n = 6; * p<0.05 difference between adiponectin-treated and control cells.

Adiponectin down-regulated significantly the constitutive mRNA expression of IL1β at 4 h and 24 h, as analyzed by real-time PCR (Fig. 2A). Moreover, adiponectin inhibited significantly the mRNA expression of IL6 at 24 h and of IL8 at 4 h (Fig. 2A). Interestingly, anti-inflammatory mediators were also subject to regulation by adiponectin: After an initial adiponectin-induced down-regulation of IL10 mRNA at 4 h and 8 h, the levels of this anti-inflammatory molecule were significantly increased at 24 h. In addition, the HMOX1 expression was significantly enhanced by adiponectin at 4 h and 8 h (Fig. 2B). In summary, these data suggest that adiponectin regulates the expression of pro- and anti-inflammatory mediators in oral epithelial cells.

### Effect of adiponectin on the LPS-stimulated nuclear translocation of NFκB

**Figure 3:**
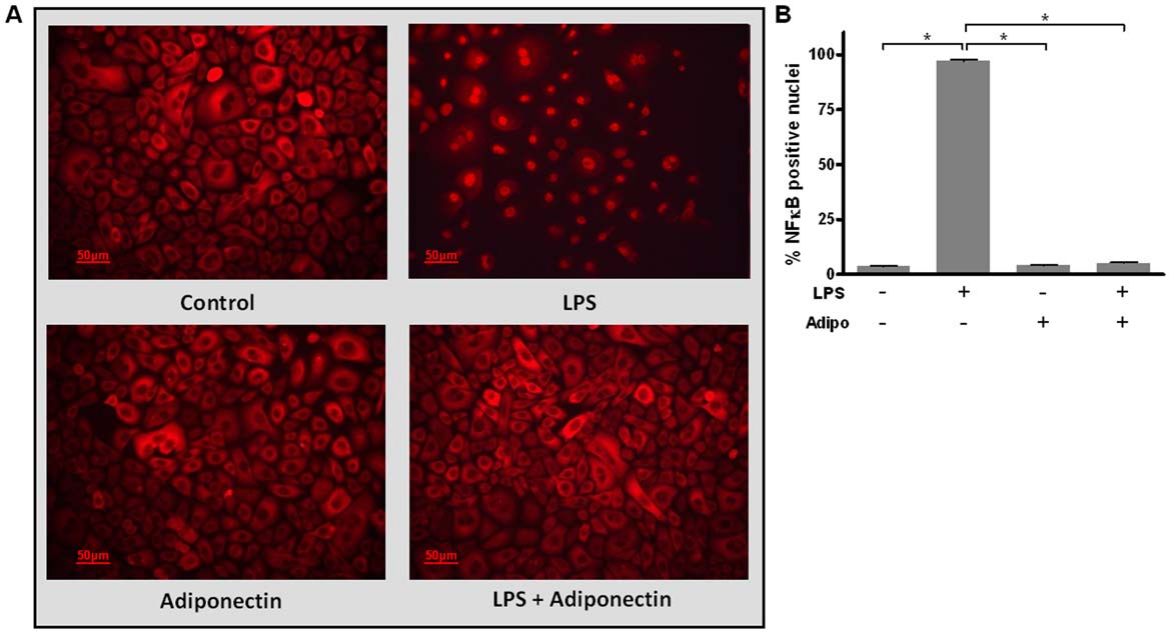
Effect of lipopolysaccharide and/or adiponectin on NFκB signaling. Effect of lipopolysaccharide (LPS; 2 µg/ml) and/or adiponectin (Adipo; 1 µg/ml) on the nuclear translocation of NFκB at 2 h. NFκB visualized by immunofluorescence (A). Quantitative analysis of immunofluorescence samples; evaluation as % NFκB positive nuclei (B).

As shown in Fig. 3A and B, LPS from *P. gingivalis* induced a pronounced nuclear translocation of NFκB at 2 h, whereas no such effect was observed for adiponectin. Interestingly, adiponectin abolished the LPS-stimulated NFκB translocation, as visualized by immunofluorescence (Fig. 3A and B).

### Effects of adiponectin on the LPS-induced expression of pro- and anti-inflammatory cytokines

**Figure 4:**
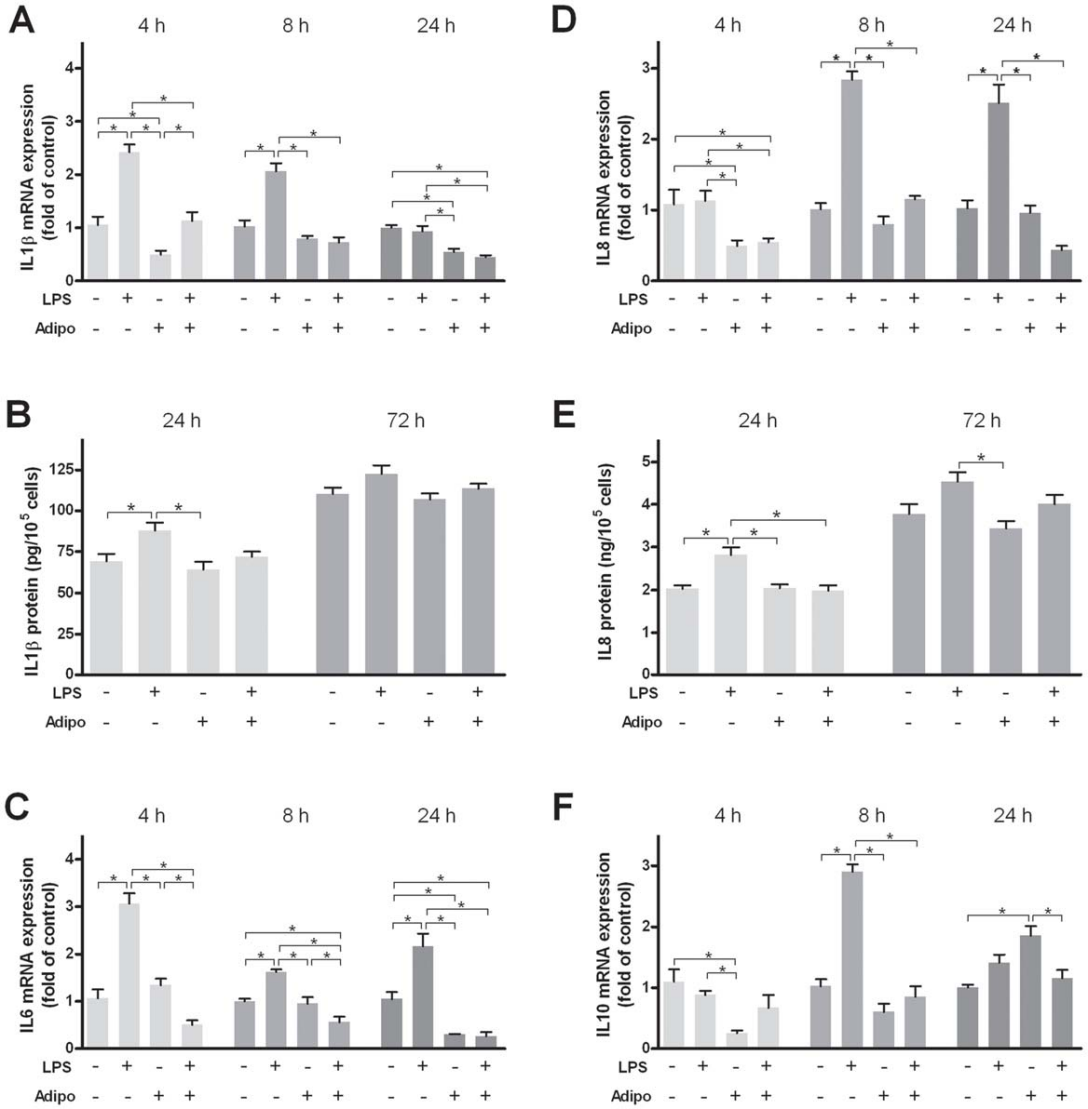
Interactions of lipopolysaccharide and adiponectin on pro- and anti-inflammatory mediators. Effects of lipopolysaccharide (LPS; 2 µg/ml) and/or adiponectin (Adipo; 1 µg/ml) on the mRNA expression of IL1β (A), IL6 (C), IL8 (D), and IL10 (F) at 4 h, 8 h, and 24 h and on the protein level of IL1β (B) and IL8 (E) in supernatants at 24 h and 72 h. Mean ± SEM; n = 6; * p<0.05 difference between groups.

LPS caused a significant up-regulation of the IL1β mRNA expression at 4 h and 8 h but had no stimulatory effect on this cytokine at 24 h (Fig. 4A). Interestingly, adiponectin significantly down-regulated the LPS-induced IL1β expression at 4 h and 8 h, and also led to a significant decrease in the constitutive IL1β expression at 4 h and 24 h (Fig. 4A). Similar findings were observed at protein level at 24 h, as determined in the supernatants by ELISA (Fig. 4B). Furthermore, LPS significantly increased the IL6 mRNA expression at 4 h, 8 h, and 24 h. As observed for IL1β, IL6 was significantly down-regulated by adiponectin in LPS-stimulated cells (Fig. 4C). Although adiponectin did not affect the constitutive IL6 mRNA expression at 4 h and 8 h, this adipokine caused a significant reduction of the constitutive IL6 expression at 24 h (Fig. 4C). The chemokine IL8 was also significantly up-regulated by LPS at 8 h and 24 h (Fig. 4D). The LPS-induced IL8 mRNA expression was significantly reduced by adiponectin at 8 h and 24 h. Additionally, the constitutive mRNA expression of IL8 was down-regulated by adiponectin at 4 h (Fig. 4D). The transcriptional data on IL8 were paralleled by findings at protein level (Fig. 4E). LPS also up-regulated significantly the mRNA expression of the anti-inflammatory cytokine IL10 at 8 h (Fig. 4F). However, adiponectin abrogated the stimulatory effects of LPS on IL10 expression at this time point. Moreover, adiponectin inhibited significantly the constitutive IL10 expression at 4 h and stimulated significantly the constitutive expression of IL10 mRNA at 24 h (Fig. 4F). In summary, these results suggest that adiponectin may dampen inflammatory processes in periodontitis by regulation of pro- and anti-inflammatory cytokines.

### Effects of adiponectin on the LPS-induced up-regulation of MMPs

**Figure 5:**
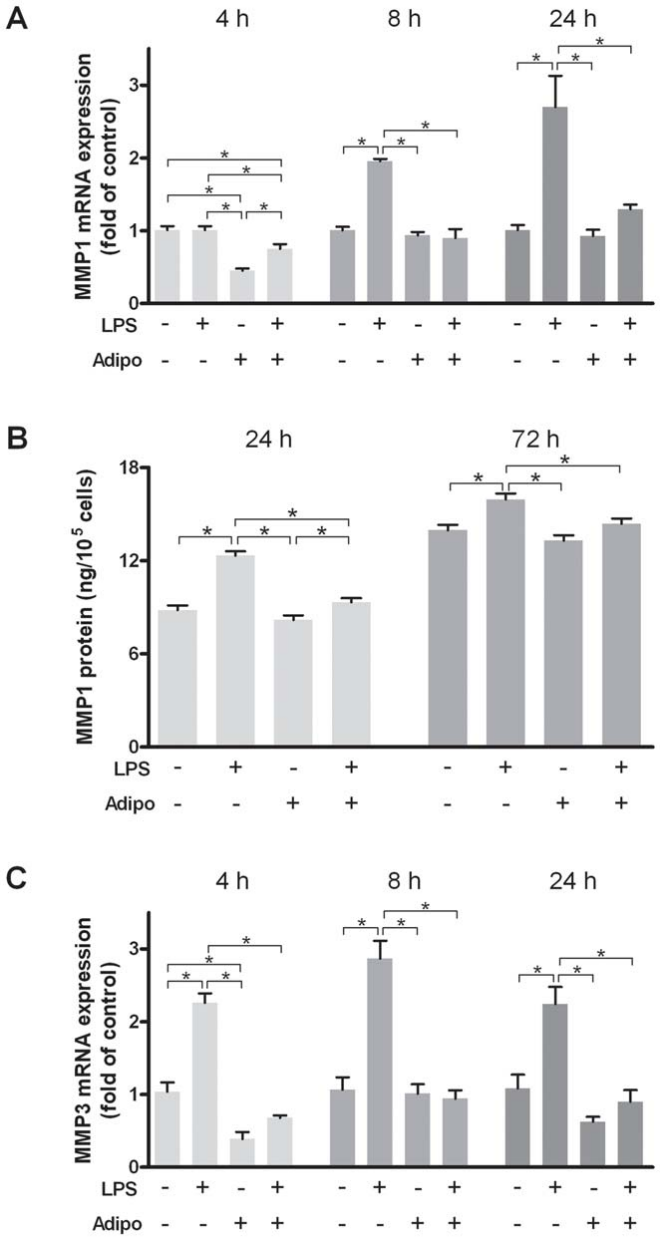
Interactions of lipopolysaccharide and adiponectin on matrix metalloproteinases. Effects of lipopolysaccharide (LPS; 2 µg/ml) and/or adiponectin (Adipo; 1 µg/ml) on the mRNA expression of MMP1 (A) and MMP3 (C) at 4 h, 8 h, and 24 h and on the protein level of MMP1 (B) in supernatants at 24 h and 72 h. Mean ± SEM; n = 6; * p<0.05 difference between groups.

LPS also affected the production of the matrix-degrading proteases MMP1 and MMP3 in epithelial cells (Fig. 5A–C). In the presence of LPS, MMP1 and MMP3 were significantly up-regulated at 8 h and 24 h and at 4 h, 8 h, and 24 h, respectively (Fig. 5A and C). At all time points, adiponectin diminished significantly the LPS-induced mRNA expression of MMP1 and MMP3 (Fig. 5A and C). The stimulatory effect of LPS on MMP1 and the inhibition of the LPS action by adiponectin were also observed at protein level (Fig. 5B). Our data suggest that adiponectin exerts matrix-protective effects by down-regulation of MMPs.

### Effects of LPS and/or adiponectin on proliferation, cell viability and wound fill rate

**Figure 6:**
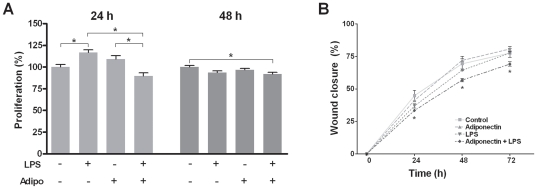
Interactions of lipopolysaccharide and adiponectin on proliferation and wound closure. Effects of lipopolysaccharide (LPS; 2 µg/ml) and/or adiponectin (Adipo; 1 µg/ml) on the proliferation at 24 h and 48 h (A) and wound closure over 72 h (B). Mean ± SEM; * p<0.05 difference between groups (A) and difference between cells simultaneously treated with LPS and adiponectin, and control (A).

Although the epithelial cell proliferation was not strongly affected by LPS and/or adiponectin, the stimulatory effect of LPS was significant at 24 h (Fig. 6A). Interestingly, the LPS-induced increase in epithelial cell proliferation was significantly counter-regulated by adiponectin at this time point. These findings suggest that adiponectin may inhibit the formation of pocket epithelium in the presence of periodontal infection. Furthermore, our experiments revealed, that LPS significantly reduced the percentage of viable cells over a time period of 72 h and that the LPS-induced decrease in cell viability was significantly abolished in the presence of adiponectin (Table 2), indicating that adiponectin may protect against infection-induced damage of epithelial cells and, thereby, increased permeability of the epithelial barrier. Next we studied whether LPS and/or adiponectin modulated the wound closure in an in-vitro wound healing assay over 3 days. The wound closure in cell cultures treated either with LPS or adiponectin alone was not significantly different from that of control cells. However, when cells were simultaneously exposed to LPS and adiponectin, the wound closure was significantly delayed as compared to control (Fig. 6B). These findings suggest that adiponectin, when combined with LPS from *P. gingivalis*, may inhibit the wound fill rate in LPS-treated epithelial cells.

**Table 2:** Interactions of lipopolysaccharide and adiponectin on cell viability.

Cell viability (%)			
Group	24 h	48 h	72 h
Control	93.30±0.96	95.63±0.58	96.79±0.64
LPS	79.25±2.80*	84.03±0.47*	80.49±0.90*
Adipo	99.81±1.02* †	99.73±0.32* †	99.78±0.77†
LPS + Adipo	95.22±0.36†	90.97±0.30* ^†^ #	96.06±0.11^†^ #

Effects of lipopolysaccharide (LPS; 2 µg/ml) and/or adiponectin (Adipo; 1 µg/ml) on the cell viability, as assessed by trypan blue exclusion test, at 24 h, 48 h, and 72 h. Mean ± SEM; n = 6;

* p<0.05 different from control,

† different from LPS-treated cells,

# different from adiponectin-treated cells.

### Inhibition of the LPS-induced involucrin expression by adiponectin

LPS increased significantly the involucrin mRNA expression in epithelial cells at 4 h and 8 h. An LPS-induced up-regulation of involucrin was also observed at 24 h but the increase did not reach significance (Table 3). When LPS-treated cells were exposed to adiponectin, the involucrin mRNA expression was significantly reduced (Table 3). These data suggest that adiponectin may inhibit the formation of a pocket epithelium by both inhibition of the LPS-induced proliferation and, additionally, the LPS-stimulated epithelial differentiation.

**Table 3:** Interactions of lipopolysaccharide and adiponectin on involucrin and growth factors.

Target gene	Group	4 h	8 h	24 h
Involucrin	LPS	2.91±0.12*	4.86±0.34*	1.58±0.24
	Adipo	0.87±0.13†	2.50±0.21* †	0.97±0.23
	LPS + Adipo	0.89±0.21†	2.02±0.14* †	0.69±0.10†
TGFβ1	LPS	1.06±0.07	0.77±0.07	0.83±0.19
	Adipo	0.28±0.04* †	1.03±0.06	0.52±0.09*
	LPS + Adipo	0.96±0.14#	0.75±0.04* #	0.84±0.05
KGF	LPS	0.63±0.16	2.01±0.11*	0.59±0.03*
	Adipo	0.19±0.04* †	0.64±0.06* †	1.02±0.10†
	LPS + Adipo	0.49±0.09*	0.68±0.08†	0.41±0.05* #

Effects of lipopolysaccharide (LPS; 2 µg/ml) and/or adiponectin (Adipo; 1 µg/ml) on the mRNA expression of involucrin, TGFβ1, and KGF at 4 h, 8 h, and 24 h expressed as fold of control. Mean ± SEM; n = 6;

* p<0.05 different from control,

† different from LPS-treated cells,

# different from adiponectin-treated cells.

### Regulation of TGFβ1 and KGF by adiponectin and/or LPS

Although the constitutive TGFβ1 expression was not significantly regulated by LPS, adiponectin caused a significant TGFβ1 down-regulation at 4 h and 24 h (Table 3). Adiponectin did not significantly affect TGFβ1 levels in LPS-treated cells (Table 3). Furthermore, LPS significantly up-regulated KGF at 8 h and significantly down-regulated this growth factor at 24 h. Although adiponectin abolished the LPS-increased KGF expression at 8 h, this adipokine had no significant influence on the LPS-down-regulated KGF expression at 24 h. In addition, adiponectin decreased significantly the constitutive KGF mRNA expression at 4 h and 8 h in epithelial cells. In summary, these data suggest that adiponectin may exert inhibitory effects on KGF expression and, therefore, formation of pocket epithelium.

## Discussion

Our experiments demonstrated that LPS from *P. gingivalis*, which is considered one of the main etiological agents of periodontal diseases, elicits synthesis of pro-inflammatory cytokines and matrix-degrading enzymes and promotes proliferation and differentiation of oral epithelial cells, emphasizing the pathogenic role of this microorganism in periodontal inflammation, destruction and pocket formation. However, more importantly, our study shows that the LPS-induced effects on oral epithelial cells are counteracted by adiponectin, which is a novel finding and might, at least partially, explain how overweight and obesity can increase the risk of periodontitis.

LPS, which is a major macromolecule on the outer surface of *P. gingivalis*, has been shown to bind to TLR2 and TLR4 [8], [10]. Upon receptor engagement, LPS triggers an intracellular signaling cascade, which involves the nuclear transactivation of NFκB [8].

The gingival epithelium is the first physical barrier, which periodontopathogenic bacteria, such as *P. gingivalis*, encounter [6]. Our experiments revealed that *P. gingivalis*-LPS induces the expression and release of pro-inflammatory cytokines in oral epithelial cells, which underlines the detrimental role of this pathogen in periodontal diseases. These findings are in line with several other in-vitro studies, which have also demonstrated a stimulatory effect of *P. gingivalis*-LPS on the synthesis of these inflammatory mediators in oral epithelial cells [11], [13], [36]. In one of these studies, it was also analyzed whether LPS from *P. gingivalis* induces the release of proteases [36]. *P. gingivalis*-LPS did not affect the secretion of MMP2 and MMP9 in oral epithelial cells. Since MMP1 and MMP3 have also been implicated in periodontal destruction, we studied their expression in oral epithelial cells in response to *P. gingivalis* [37]–[39]. Interestingly, both MMPs were up-regulated by LPS, suggesting that LPS from *P. gingivalis* can promote periodontal destruction via stimulation of both inflammation and matrix-degradation. In one study, oral epithelial cells were exposed to *P. gingivalis*-derived membrane vesicles, which retain the full components of the outer membrane constituents, including LPS [12]. The expression of IL6, IL8, MMP1, and MMP3 were significantly up-regulated in the vesicle-stimulated cells, which is in accordance with our data for LPS. The most important and novel finding of our study is that the pro-inflammatory and matrix-degrading effects of LPS on oral epithelial cells were significantly reduced in the presence of adiponectin. This observation is in accordance with several studies on monocytes and macrophages [25], [32], [40]. However, periodontopathogens, such as *P. gingivalis*, will usually first encounter oral epithelial cells, which are present in the gingiva in great numbers. Therefore, our finding, that the protective actions of adiponectin are not limited to immunoinflammatory cells in the gingiva, might be of utmost importance.

A few studies have suggested that adiponectin also exerts pro-inflammatory and tissue-destructive effects [41], [42]. It has even been speculated that an initial up-regulation of pro-inflammatory mediators by adiponectin might also be the cause for the subsequent anti-inflammatory effects of this adipokine [43], [44]. Moreover, this initial pro-inflammatory effect might result from contamination of adiponectin with LPS during the manufacture process [45]. Interestingly, such an initial adiponectin-induced increase of pro-inflammatory cytokines was not observed in our study. Furthermore, IL10 has been shown to mediate some of the anti-inflammatory effects of adiponectin [25], [40], [43]. In the present study, we did not perform blocking experiments for IL10. However, the pro-inflammatory cytokines were already down-regulated by adiponectin at 4 h, when the IL10 expression was not yet increased by LPS and/or adiponectin, indicating that IL10 may not play a role in the anti-inflammatory actions of adiponectin at this early time point. Whether adiponectin mediates anti-inflammatory effects via up-regulation of IL10 at later time points, as observed at 24 h, needs to be further determined. Recently, HMOX1 has been identified as another important downstream mediator for the anti-inflammatory actions of adiponectin in primary cultures of Kupffer cells [25]. Interestingly, HMOX1 was significantly up-regulated by adiponectin in oral epithelial cells, suggesting that HMOX1 might be responsible, at least in part, for the anti-inflammatory effects of adiponectin in our study. Finally, adiponectin might interfere with the binding of LPS to its receptor because of the high binding affinity of adiponectin to LPS, suggesting an additional extracellular mode of anti-inflammatory action for adiponectin [46]. If this mechanism also contributed to the anti-inflammatory effects of adiponectin in our experiments remains to be elucidated.

In the circulation, adiponectin occurs as full-length and globular adiponectin. Full-length adiponectin exists as low, middle and high molecular weight oligomeric complexes. In addition, full-length adiponectin can be proteolytically cleaved to globular adiponectin, which consists of the C-terminal domain of the full-length protein. Both forms of adiponectin are biologically active and bind to the adiponectin receptors. Upon receptor engagement, adiponectin triggers several intracellular signaling pathways [23], [47], [48]. Different forms of adiponectin have been used in in-vitro studies, and although these forms often have similar effects, some actions are different, which could also explain controversial results among studies [47]. As mentioned above, LPS triggers an intracellular signaling cascade, which involves the nuclear translocation of NFκB [8]. Our experiments revealed that adiponectin inhibits the LPS-induced NFκB nuclear translocation in oral epithelial cells. This observation concurs with findings in other cells, where adiponectin inhibited the LPS-stimulated IκB degradation, thus preventing NFκB activation and DNA binding activity of NFκB [49]–[52]. Whether adiponectin also affects other LPS-triggered signaling pathways, such as ERK1/2, as it has been suggested, needs to be determined in further studies [52], [53].

Since epithelial cell migration, proliferation, and differentiation are critical to periodontal pocket formation in periodontitis, we also studied the actions of LPS and adiponectin on these parameters [1]. In an in-vitro wound healing assay, which mainly assesses cell migration and proliferation, adiponectin alone had no significant effect on epithelial cells. However, when these cells were exposed to adiponectin in the presence of LPS, the wound closure was significantly delayed, suggesting that adiponectin may inhibit cell migration in the presence of periodontal infection. At 1 day, adiponectin also inhibited the proliferation of oral epithelial cells exposed to LPS, suggesting that adiponectin may have, at least initially, some anti-proliferative effects in the presence of periodontal infection. Interestingly, adiponectin also reduced the LPS-induced cell proliferation and migration in a study on adventitial fibroblasts, supporting our results in oral epithelial cells [54]. The cell viability was significantly reduced by LPS but the LPS-induced decrease in cell viability was abrogated by adiponectin, indicating that adiponectin may protect against infection-induced damage of epithelial cells. However, another study did not observe *P. gingivalis*-induced changes in oral epithelial cell viability and numbers, but the controversial results could be explained by the different sources, strains and/or concentrations of LPS [11]. In studies on cancer cells, it has been shown that the anti-proliferative effect of adiponectin involves inhibition of cell cycle and activation of cell apoptosis [27], [55]. Whether *P. gingivalis*-LPS and adiponectin also affect apoptosis of oral epithelial cells remains to be examined in further studies. The epithelium acts as a protective barrier against physical, chemical, and microbial insults. In order to fulfill this function, epithelial cells undergo differentiation and express a number of structural proteins, such as involucrin [28]–[30]. Our experiments revealed that LPS from *P. gingivalis* up-regulates involucrin, whereas adiponectin counteracts the stimulatory effects of LPS on this differentiation marker, suggesting that adiponectin may prevent epithelial cell differentiation and, thereby, formation of pocket epithelium, in the presence of periodontal infection.

Since epithelial proliferation and migration are regulated by growth factors, we also studied the expression of KGF1 and TGFβ1 in oral epithelial cells. KGF1 belongs to the heparin-binding fibroblast growth factor family, binds to the epithelial-specific KGF receptor and stimulates epithelial cell proliferation and migration [56]–[58]. Moreover, KGF1 has been shown to inhibit tumor necrosis factor-α- and LPS-induced epithelial cell apoptosis [59]. Although KGF is usually expressed by connective tissues, this growth factor has also been detected in epithelial cells, where it seems to act in an autocrine manner [60], [61]. It has been hypothesized that the KGF1 up-regulation in periodontal diseases is associated with the onset and progression of periodontal pocket formation. The increased KGF1 protein production in epithelial cells may reduce apoptosis and maintain the integrity of the epithelium despite bacterial infection [62]. Although epithelial cell proliferation is a hallmark of periodontitis, the regulation of this proliferation is only partially unraveled. Our experiments revealed that LPS from *P. gingivalis* transiently up-regulated KGF at 8 h and decreased the KGF expression at 24 h. More importantly, adiponectin down-regulated significantly the constitutive KGF expression at 4 h and 8 h and also inhibited significantly the LPS-induced KGF expression at 8 h. Thus, the anti-proliferative effect of adiponectin in the presence of LPS may be, at least in part, mediated by inhibition of the KGF expression. TGFβ1 is another important growth factor, which regulates cell proliferation and migration, matrix synthesis, angiogenesis, and wound healing processes. TGFβ1 has both pro-inflammatory and immunosuppressive effects [63]. Increased TGFβ1 concentrations in gingival tissues and GCF from sites with deeper periodontal pockets have been reported [64]. In the present study, LPS had no effect on TGFβ1, and the addition of adiponectin to LPS-stimulated cells caused only minor changes in TGFβ1 expression. Thus, the anti-proliferative effects of adiponectin on LPS-treated cells do not seem to be dependent on TGFβ1.

In summary, our experiments demonstrated that adiponectin abrogates the *P. gingivalis*-LPS-induced up-regulation of pro-inflammatory cytokines and matrix-degrading enzymes as well as proliferation and differentiation of oral epithelial cells. Therefore, low levels of adiponectin, as observed in overweight and obese individuals, may confer an increased risk for periodontal inflammation, destruction and pocket formation.
